# Blood-based Markers in the Prognostic Prediction of Esophagogastric Junction Cancer

**DOI:** 10.7150/jca.44545

**Published:** 2020-04-27

**Authors:** Can-Tong Liu, Chao-Qun Hong, Xu-Chun Huang, En-Min Li, Yi-Wei Xu, Yu-Hui Peng

**Affiliations:** 1 Department of Clinical Laboratory Medicine, the Cancer Hospital of Shantou University Medical College, Shantou, Guangdong, China; 2 Precision Medicine Research Center, Shantou University Medical College, Shantou, Guangdong, China; 3 Department of Oncological Laboratory Research, the Cancer Hospital of Shantou University Medical College, Shantou, Guangdong, China; 4 Department of Biochemistry and Molecular Biology, Shantou University Medical College, Shantou, Guangdong, China

**Keywords:** esophagogastric junction cancer, blood-based marker, prognosis

## Abstract

Esophagogastric junction cancer poses a great threat to human beings both in western countries and East Asia, especially in China and Japan, and its incidence has increased during recent decades. The 5-year survival rate of esophagogastric junction cancer is quite poor compared with that of other gastric cancer sites. Until now, the traditional TNM staging system has been widely used in clinical practice for prognosis. However, the TNM system is based on pathology after surgical resection or radiology using CT and MRI, not on blood markers. Evidently, some research has been reported concentrated on the prognostic value of blood-based markers with the character of non-invasive and non-radioactive in EJA. Hematologic, biochemical and coagulation parameters could be obtained from clinical data and utilized to analyze their prognostic values. Tumor-associated antigens, microRNAs and circulating tumor cells have also been reported in EJC prognosis. In this article, we review research focused on blood-based markers to evaluate their prognostic value in esophagogastric junction cancer, especially its main subtype adenocarcinoma.

## Introduction

Esophageal cancer and gastric cancer are two common malignant diseases, ranking sixth and ninth, respectively, in the incidence of cancers worldwide 1. Esophagogastric junction cancer (EJC), whose main type is adenocarcinoma (EJA), is a malignant tumor with the center located within a 10-centimeter distance between the esophagus and stomach. In recent decades, the incidences of these two cancers have decreased, but EJC has instead increased in both East Asia and western countries 2. EJC was first described by Siewert in 1998 3 and has unique biological characteristics. Gastroesophageal reflux disease and *Helicobacter pylori* are associated with the increased risk of suffering from EJC 4, 5, and Barrett's esophagus (BE) is recognized as the precancerous lesion of adenocarcinoma in western countries 6.

The clinical manifestation of most patients suffering from EJC is dysphagia, which only becomes symptomatic at an advanced stage. With asymptomatic characteristics and the unpopularity of endoscope screening for early-stage EJC, Chinese patients tend to be diagnosed in the advanced stage 7. Serosal invasion, lymph node metastasis and hematogenous recurrence are more likely to appear in EJC compared with the distant gastric cancer 8, which might be the reason why the 5-year overall survival (OS) rates of advanced stage EJC patients, who had undergone curative therapy, is less than 30%^9^, lower than that of cancers occurring in other sites of the stomach. Although chemoradiotherapy does assist in improving the survival time in locally advanced EJC, the 5-year OS rates still remain low, ranging from 23% to 38% 10.

The American Joint Committee on Cancer Eighth Edition Cancer Staging Manual is widely used to predict the probable survival rate of esophageal cancer and EJC 11. When staging EJC, tumors with centers no more than 2 centimeters into the gastric cardia are staged as esophageal carcinomas, while those with more than 2 centimeters are staged as gastric cancers. The latter used to be named gastric cardia cancer, the Siewert type III. The traditional TNM staging system, containing invasive depth, regional node metastasis and distant metastasis, is based on pathology after surgery or endoscopy, or computerized tomography and magnetic resonance imaging. When determining whether distant metastasis occurs, positron emission tomography is usually used. However, not included is any information from blood, an easily accessed, non-invasive and non-radioactive source.

Blood can be used to evaluate inflammation and nutritional status by testing its contents. After centrifugation, evaluation in serum and plasma of tissue function, such as liver and renal function, and coagulation function, can be determined. In the case of tumors, tumor-associated RNAs, proteins or cells, recognized as tumor-associated markers, will be released into the peripheral blood and can be utilized to assist in diagnosis and determination of the prognosis of cancers 12. Positive detection of tumor candidates might indicate the existence of cancers, and their different concentrations might lead to different survival times. Recent concerns have arisen in the area of prognostic analysis of EJC based on blood-based markers. Here, we review relevant literatures on the value of blood-based markers for prognostic prediction in EJC.

## Hematologic Parameters

The complete blood cell count (CBC) is a common method for evaluating inflammation and nutritional status. It can be completed in a few minutes after sampling without a complex and expensive facility. Therefore, its use is widespread in community hospitals. In the last few years, inflammation has been accepted as a hallmark in cancer progression and prognosis, and it can be evaluated with blood parameters, such as leukocytes 13. Some parameters, including neutrophils and lymphocytes, have been discovered to be prognostic factors in many cancers 14. Erythrocytes and platelets are generated from marrow, and their related parameters can show the function of marrow hematopoiesis, hinting at potential prognostic value of tumors.

**Figure 1:**
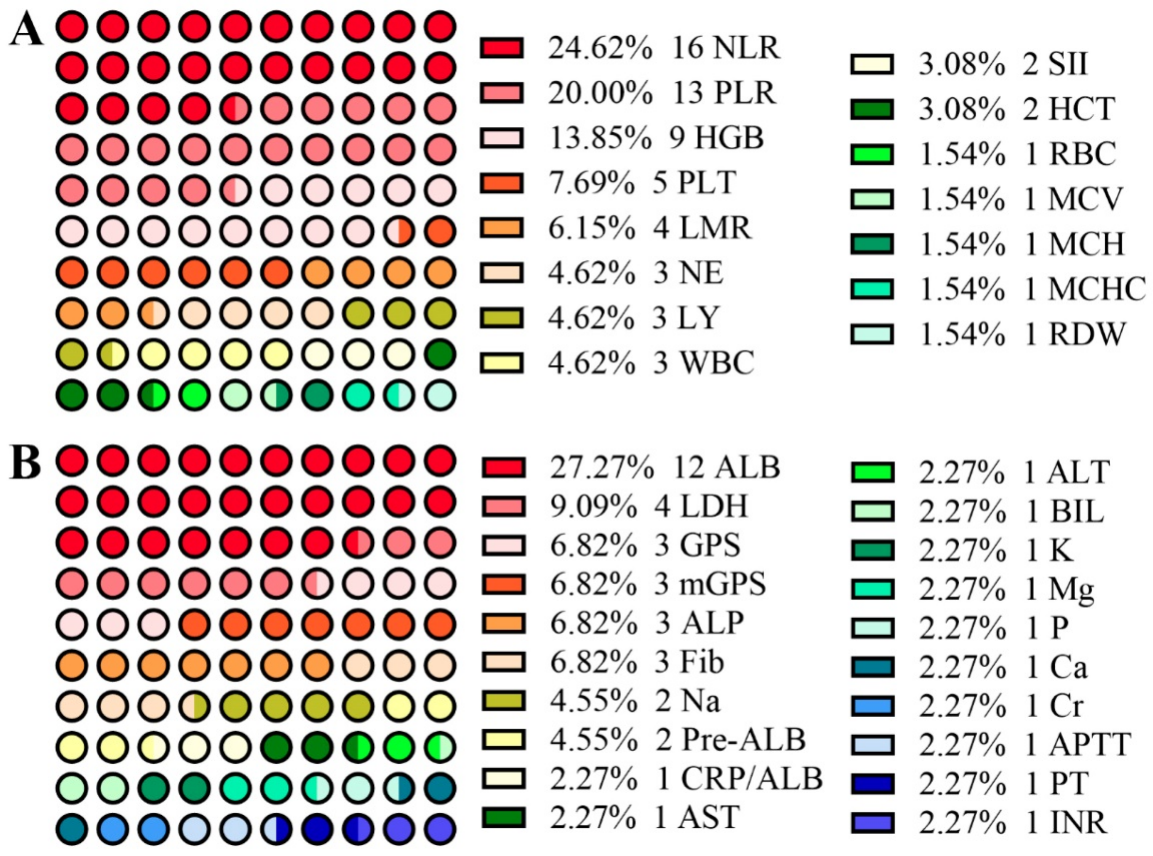
Dot diagrams of the number of studies involving blood-based hematologic parameters (A), and biochemical and coagulation parameters (B).

From Figure 1A, among research involving CBC, the neutrophil-lymphocyte ratio (NLR) and platelet-lymphocyte ratio (PLR) are two of the most popular criteria in predicting prognosis of EJC. As shown in Table 1, increased NLR is one of the most frequently observed markers in EJC 15-18. With cutoff values varying from 1.84 - 4.00, the NLR might act as a potential marker in predicting the survival rate of patients with EJC 19-26, especially for patients who have undergone surgery. The NLR has been found to be correlated to tumor size 21, age 22 and T stage 24. Although most of these studies involved a small sample size of patients, Wang et al. conducted a large-sample study (435 EJA patients in 1498 gastroesophageal adenocarcinoma patients) and showed that pretreatment NLR, as a continuous variable, can predict cancer-specific survival (CSS) independently in resectable EJA patients regardless of whether or not patients received neoadjuvant therapy 24. Moreover, Zhang et al. found that a NLR value higher than 3.5 independently led to a poor overall survival of Siewert type II/III EJA (355 EJA patients) 21. A larger study (611 EJA patients) performed by Zhang et al. suggested that NLR was associated with CSS, but does not play a vital role in predicting CSS of Siewert type II/III EJA^27^. Among these studies, NLR was correlated with T and N stages 24 and patients with NLR higher than 3 had a short overall survival time in stages IIB and III 22. Therefore, it is important to further explore the predictive value of the NLR for predicting prognosis of EJA in a large-sample and multi-center study.

Thrombosis is frequent in cancer patients, resulting in high morbidity and mortality 28, and platelets participate in the process. Platelets coordinate in the immune system and affect cancer-related inflammation by changing the activation status of the endothelium and recruiting leukocytes to tumor sites 29. It is reported that lymphocytes are vital for cancer immune-surveillance and immune-editing 30. PLR, a combination of platelets and lymphocytes, has been found to be a prognostic factor in different cancers 31, 32. In EJA patients receiving neoadjuvant therapy, Messager et al. found that an elevated PLR (PLR > 192) is associated independently with decreased disease-free survival (DFS; hazard ratio [HR] = 2.85, 95% CI: 1.54 - 5.26, *p* = 0.001) and overall survival (OS; HR = 2.47, 95% CI: 1.21 - 5.01, *p* = 0.012) 33. Another study suggested a significant *p-*value of PLR (*p* = 0.038) in univariate analysis, but failed to further evaluate the independent probability 34. Nevertheless, Zhou et al. conducted a retrospective study on EJA patients who underwent radical surgery to find that it was the higher preoperative lymphocyte-monocyte ratio (LMR), not NLR or PLR, that independently predicts poor OS 35.

There were a few studies focusing on the association between absolute neutrophil (NE), lymphocyte (LY) or platelet (PLT) counts and EJC prognosis 26, 34, 36-38. However, only Fuchs et al. found that abnormally low blood levels of LY (HR = 1.31, 95% CI: 1.05 - 1.63, *p* = 0.0015) and high levels of NE (HR = 1.52, 95% CI: 1.17 - 1.99, *p* < 0.0001) were both candidates for predicting risk of EJC patients who underwent 4-month, first-line chemotherapy (platinum and/or fluoropyrimidine with or without an anthracycline) 37. When combining these three parameters, a systemic immune-inflammation score (SII) has emerged, calculated by using a formula (SII = NE×LY/PLT), first described in 2014 to explore its prognostic value in hepatocellular carcinoma 39. Jomrich et al. also introduced it for EJA and found that a higher SII contributes to poor OS and DFS in EJA patients who underwent esophagectomy with or without receiving neoadjuvant treatment 40.

With the occurrence of gastrointestinal bleeding, injury or aplastic anemia, RBCs will decrease, as well as hemoglobin (HGB). In a multicenter randomized trial including 248 EJC patients, an HGB lower than 110 g/l has been excluded from the baseline prognostic model, although it showed significantly poor quality of life 38. However, another study from China, conducted by Zhu et al., found that an HGB over 130 g/l might be a protective marker for EJA, but not in other gastric cancers 41, which was not in accordance with a previous study involving only stage I and II patients 42. Thus, a hierarchical analysis in different stages is provably needed. When turning to HGB- or RBC-related factors, few studies have been reported for EJC. Jomrich et al. evaluated the prognostic value of preoperative mean corpuscular volume (MCV), mean corpuscular hemoglobin (MCH), mean corpuscular hemoglobin concentration (MCHC), and red blood cell distribution width (RDW) for patients with resectable EJC. For all patients, elevated MCV, MCH, and MCHC remained highly associated with reduced OS and DFS, and Cox regression analysis showed they could be independent prognostic factors in all EJC patients, but only MCV made sense in both OS and DFS in patients who were given neoadjuvant treatment 43. In consideration of the delicate relationship between MCH and alcohol consumption in ESCC 44, the potential mechanism between MCV and alcohol in EJC might be another focus in the future.

## Biochemical and Coagulation Parameters

Biochemical detection is popular in clinical practice. For example, high levels of alanine aminotransferase (ALT), aspartate aminotransferase (AST) and hypoalbuminemia usually indicate impairment of liver function. Hypoalbuminemia might result from reduced consumption. As mentioned before, the characteristic symptom of EJC is dysphagia, which will lead to a smaller diet and ultimately decreased albumin (ALB). The more serious the dysphagia, the lower the serum ALB. Thus, ALB might be a potential predictive marker for EJC. In fact, from Table 2, setting 35 g/l as the cutoff value, four studies all showed that the low preoperative albumin, the most popular research subject (Figure 1B), can be a potentially independent marker for predicting poor survival of EJC^37^, ^45^-^47^. As an acute-phase protein with shorter half-life (about 1.9 days) than ALB, pre-albumin, a 54 kDa protein, has become another focus of research. Han et al. and Zhang et al., from one research team, showed that a high level of pre-albumin could predict longer OS in EJA patients with Siewert type II and III who received gastrectomy 21, 48.

A team from the Royal Marsden Hospital (RMH) conducted three randomized, controlled trials, and built a prognostic model using performance status, liver metastases, peritoneal metastasis, and alkaline phosphatase (ALP), to assess survival time in patients with locally advanced or metastatic EJA patients who underwent different chemotherapies 38. In this RMH prognostic system, an ALP over 100 U/l hinted at poor survival time and quality of life. It also correlated with a significantly reduced probability of tumor response to chemotherapy. Another study from the Yale Cancer Center recruited more than 1,000 patients with gastric cancer or EJC and collected 41 baseline factors, including biochemical and coagulation parameters 37. They found that high ALP, lactate dehydrogenase (LDH) and AST levels, and low albumin and sodium levels were independent markers for predicting poor OS. Meanwhile, another prognostic model was built based on 7 blood-based markers and other factors besides peritoneal metastases and Eastern Cooperative Oncology Group performance scores. The patients listed in these two models were ones with advanced cancers who received chemotherapy. However, there are fewer models based on blood markers in early-stage patients or patients with resectable EJA.

C-reactive protein (CRP) is an acute protein that rises sharply in plasma when the body is infected or damaged due to any type of inflammation. After activating complement, it can strengthen phagocytosis by phagocytes to play a complementary role, and clears away pathogenic microorganisms that invade the body and tissue cells that are damaged, necrotic and apoptotic. Combining CRP and ALB, CRP/ALB and Glasgow Prognostic Score (GPS) has been reported to assess EJC survival. Kudou et al. found that it was the CRP/ALB, but not GPS, that was strongly associated with poor OS in patients who underwent surgery for EJC 49. Patients with high T stages or N stages preferred to contain a larger CRP/ALB which indicated poor RFS and OS. Compared with GPS, patients with a normal CRP level (≤ 1.0 mg/dl) regardless of albumin were given a modified GPS (mGPS) of 0^50^. Jomrich et al. thought that post-neoadjuvant therapy mGPS is highly associated with OS and DFS in patients suffering from neoadjuvantly-treated EJA (HR = 1.72, 95% CI: 1.10 - 2.67 for OS; HR: 1.65, 95% CI: 1.08 - 2.50 for DFS) 46. A research from China also determined its prognostic value in predicting OS and DFS in EJA patients with resection 51. Park et al. suggested that mGPS might be an independent marker for survival in patients with EJA (163 out of 203 participants, including gastric cancer) undergoing palliative self-expandable metallic stent insertion (HR = 1.24, 95% CI: 1.03 - 1.49) 52.

## Combination of CBC and Biochemical or Coagulation Parameters

The controlling nutritional status (CONUT) score is calculated from the serum albumin, total cholesterol, and absolute lymphocyte count 53, and better predicts survival than NLR and GPS in gastric cancer. However, it might not be a significant independent prognostic marker in EJA patients after surgery 49. Due to the small amount of research, a further study concentrating on CONUT scores to evaluate the prognostic value of EJA is needed.

Fibrinogen is a protein involved in clotting and thrombosis, and synthesized by the liver 54. Hyperfibrinogenemia has been seen to correlate with cancer progression and poor survival in colon cancer 55. In limited EJC research, there has been little concern about fibrinogen alone. A novel scoring system, denoted F-NLR, has recently aroused some attention. Patients with both hyperfibrinogenemia (≥ 3.09 g/l) and high NLR (≥ 1.89) were given a score of 2, while ones with neither hyperfibrinogenemia nor high NLR were given a score of 0.

As shown in Table 3, both studies acquired the same results in which F-NLR could be an independent factor for predicting OS of EJA patients 19, 56. Cong et al. conducted a training-validation cohort study and found the area under the receiver operating characteristic curve of F-NLR in predicting the survival of EJC was 0.717 (95% CI: 0.664 - 0.770), slightly higher than that of TNM staging (0.700; 95% CI: 0.646 - 0.754), although there was no statistical difference^19^. When stratified by pathological TNM staging, the OS of EJA patients with F-NLR 2 was poor compared with that of F-NLR 0 or 1 both in stages I - II and in stages III (all *p* < 0.001 in the combined set). In addition, Tianxing et al. found that F-NLR was associated with tumor size and TNM stage (both *p* < 0.01) 56.

First described by Pennsylvania researchers 57 and revised by Japanese researchers 58, the prognostic nutritional index (PNI) is another parameter containing CBC and biochemical indices. It can be calculated from the serum albumin concentration (g/l) plus five times the absolute lymphocyte counts (×10^9^/l). It can mirror malnutrition status due to the impaired digestive function, such as dysphagia and loss of appetite. Four studies included PNI (Table 3) 21, 26, 48, 59, but only Urabe et al. was able to show that preoperative PNI is independently associated with OS and relapse-free survival (HR = 0.62, 95% CI: 0.47 - 0.82, p < 0.001; HR = 0.60, 95% CI: 0.46 - 0.78, p < 0.001, respectively) 59 in 1363 patients who underwent surgery with gastric cancer with a small sample size of 87 EJA patients. When stratifying PNI into four groups in which patients with PNI larger than 51.9 in the fourth quartile, the authors found that constituent ratios of PNI differed in different T stages and N stages. Thus, a definite relationship between PNI and EJA survival still remains to be shown. Wang et al. tried to combine the albumin-to-globulin ratio (AGR) and PNI to establish an innovative system to estimate its prognostic value in Siewert type III EJA, and found that AGR-PNI is associated with age, tumor size, NLR and PLR (all *p* < 0.05), serving as an independent predictor for OS of EJA patients^60^. Although there was no statistically significant relationship between AGR-PNI and pathological TNM stage (*p* = 0.607), patients with AGR-PNI 1 or 2 had better OS rates in stages I+II and III than that with AGR-PNI 3.

## Tumor-Associated Circulating Materials

External and internal antigens stimulate our immune system to secrete antibodies 61. Cancer can express and release tumor-associated antigens into the circulating environment, so detection of their serum levels should assist in estimating the occurrence of malignancy, response to therapy and prognosis. Carcinoembryonic antigen (CEA) and carbohydrate antigen 19-9 (CA19-9) have been utilized for several decades as major serum tumor markers for gastrointestinal cancers. It is reported that elevated preoperative serum CEA and CA19-9 correlate with poor survival in pancreatic cancer 62. Tokunaga et al. tried to evaluate the prognostic value of CEA and CA19-9 in EJA 63. As a result, both them were found to be associated with depth of invasion and lymph node metastasis (all *p* < 0.05) and a high level of both could imply an advanced stage. However, in univariate and multivariate analysis, only CA19-9 served as a useful prognostic factor in patients with EJA (for CSS: HR = 3.89, 95% CI: 1.41 - 10.33; for OS: HR = 2.43, 95% CI: 1.03 - 5.35). Recently, a review highly commented the value of autoantibodies in the detection of esophageal cancer and EJA 64, but there lacks related studies using autoantibodies to discuss their accuracy in predicting survival time of EJA patients.

Tumorigenesis and metastasis usually partner with angiogenesis, which relies on both angiogenic and growth factors 65, 66. Using enzyme-linked immunosorbent assay, Park et al. initially detected the serum levels of several preoperative angiogenic factors, including vascular endothelial growth factor A (VEGF-A), fibroblast growth factor 2 (FGF2), epidermal growth factor (EGF) and hepatocyte growth factor (HGF), in patients with gastric cancer and EJA who underwent gastrectomy or esophagogastrectomy 67, and built an adjusted total value (ATV) uniting four factors. When these four factors were taken into consideration, multivariate analysis showed that only VEGF-A was a statistically significant independent prognostic factor for OS (*p* = 0.028) while ATV remained a powerful factor (*p* = 0.013) in another model taking into account margin status, tumor size, T category, N category and ATV. Bevacizumab is a monoclonal antibody that can inhibit VEGF, and is used for treating various metastatic cancers, including metastatic colorectal cancer and non-small-cell lung cancer. Thus, the potential of bevacizumab united with platinum in advanced EJA might be a good combination to improve survival.

Messenger RNA (mRNA) is transcribed from DNA and is translated into protein, evoking an opinion that they appear earlier than the tumor-associated proteins. Using quantitative real-time polymerase chain reaction, Qiao et al. suggested that enhanced cytokeratin 19 and CEA mRNA levels are related to lymph node metastasis. Increased pre-cytokeratin 19 and CEA mRNA levels were independent prognostic factors for OS in gastric cardia cancer patients receiving surgery 68. As noncoding 17- to 25-nucleotide-long RNA, microRNA has been seen as a new type of marker for numerous diseases, and plays vital roles in tumorigenesis, metastasis and prognosis 69. Yu et al. investigated the expression of microRNA and identified a five-microRNA signature, including hsa-let-7a, hsa-miR-221, hsa-miR-137, hsa-miR-372, and hsa-miR-182, as a novel independent prognostic factor in non-small-cell lung cancer patients 70. In the EJA field, Odenthal et al. showed, in 50 patients with local advanced EJA who underwent neoadjuvant therapy followed by surgical resection, that 122 microRNAs were differentially expressed between healthy volunteers and EJA participants 71. They indicated that high miR-302c and low miR-222 expression were significantly correlated with better OS. These two studies based on blood-based RNA verify the feasibility of using tumor markers in blood for predicting survival of EJA patients.

Circulating DNA or RNA methylation test is a research hotspot in the recent year in different cancers, such as colorectal cancer 72, hepatocellular carcinoma 73, breast cancer 74 and so on. When it came to EJC, Guo et al. detected the aberrant methylation status of long coding RNA LOC100130476 in peripheral white blood cells in three regions, different parts in exon or intron 75. Patients with region 1 (located in exon 1: from +245 to +413 bp) hypermethylation of LOC100130476 revealed significant poorer 5-year survival rates compared with those with region 1 unmethylation of the marker (P < 0.05). The Cox multivariate analysis showed that the methylation of region 1 might be an independent prognostic marker of gastric cardia adenocarcinoma.

Circulating tumor cells (CTCs), which can be derived from the primary tumor and enter into the circulation with the potential for metastasis, are another target of intense research in cancer, especially in advanced cancers. Among patients with metastatic EJA, Kubisch et al. isolated CTCs from peripheral blood of 62 patients (25 patients with EJA) and detected their mRNA levels 76. Results showed that the presence of CTCs was a predictor for OS and progression-free survival, and the mRNA transcripts were associated with tumor survival.

## Conclusion and Perspectives

Prognosis of esophagogastric junction cancer is poor. Only the traditional TNM staging system is utilized to evaluate the prognosis and treatment decision. Novel markers are urgently needed for assistant. Among we reviewed here, NLR, a popular object of study, is widely seen as a potential prognostic predictive marker. When combined with fibrinogen, F-NLR, functioned as another prognostic marker, was verified by two research teams 19, 56. The limitation of small account and single center indicated the requirement of more study. When it turns to biochemical indices, albumin and LDH might act as meaningful markers in predicting survival time of EJC.

Epstein-Barr (EB) virus, a gamma-herpesvirus, is found to be related to several diseases, such as infectious mononucleosis 77, Burkitt's lymphoma 78 and nasopharyngeal carcinoma 65. EB virus also infect the gastric epithelial cell, might leading to gastric carcinoma, which takes a nine percent in all gastric cancers 79. Although Wang et al. thought that EB virus could be associated with esophageal squamous cell carcinoma^80^, most of other research hold the same view of no pertinence in esophageal carcinogenesis 81, 82. Genitsch et al. found a low positive detection of EB virus-encoded small RNAs in tumor samples of EJC patients 83. However, the detection in peripheral blood of EJC patients is absent. Thus, it is urgently needed to explore the association between circulating EB virus antigen, antibodies or RNA and EJC.

The purpose of this review is to illuminate recent work on the predictive value of blood-based markers for prognosis in EJC. If cancer-related RNAs, proteins and cells can be taken into consideration, the accuracy for determining EJC prognosis can be enhanced. The methylation of gene might be a novel and hotspot. Moreover, related research should be completed besides the concise mechanism which is needed for elucidating how they work on the development of EJC. Most of the enrolled studies focused on the pretreatment blood markers, but not in the post-treatment fields. With the characteristics of low-cost and minimally invasive techniques, after additional verification, blood-based markers might brighten the future of treatment options for EJC.

## Figures and Tables

**Table 1:** Blood-based hematologic parameters in EJC prognosis

Variables	Authors	Number of EJC Patients	Cutoff Values	Survival Types	Hazard Ratio	95% Confidence Interval	*P-*Value
NLR	Cong X^19^	129	1.84	OS	1.820	1.316-2.517	^a^<0.001
	Conway AM^22^	316	3	TTP	1.48	1.09-2.03	0.013
				OS	1.56	1.15-2.11	0.005
	Custodio A^20^	155	4	OS	1.2086	1.0366-1.4091	0.0155
			8	OS	1.4598	1.1177-1.9064	0.0055
	Grenader T^25^	227	3	OS	1.67	1.45-1.93	<0.001
	Jagadesham VP^34^	105	2.78	MS	-	-	^d^0.061
	Jomrich G^40^	320	2.07	OS	-	-	<0.05
				DFS	-	-	<0.05
	Kudou K^49^	59	2.26	OS	3.069	1.420-7.157	^a^0.0041
	Noble F^26^	138	2.5	OS	1.191	1.092-1.298	<0.0001
				DFS	1.070	0.958-1.194	0.230
	Tianxing G^56^	129	1.89	OS	0.985	0.669-1.388	0.930
	Urabe M^59^	87	con	OS	0.97	0.89-1.07	0.56
				DFS	1.01	0.92-1.10	0.87
	Wang SC^24^	435	con	CSS	1.10	1.05-1.13	<0.0001
	Wang Y^60^	215	2.2	OS	1.118	0.805-1.550	^b^0.506
	Yuan D^23^	327	5	OS	2.551	1.847-3.524	<0.0001
				DFS	2.743	2.073-3.630	<0.0001
	Zhang JW^27^	611	2.22	CSS	1.00	0.94-1.07	^b^-
	Zhang L^21^	355	3.5	OS	2.303	1.617-3.280	0.000
	Zhou WJ^35^	309	1.697	OS	-	-	^b^>0.05
PLR	Cong X^19^	129	110	OS	1.238	0.807-1.900	0.327
	Jagadesham VP^34^	105	158	MS	-	-	^d^0.038
	Jomrich G^40^	320	146.8	OS	-	-	<0.05
				DFS	-	-	<0.05
	Kudou K^49^	59	165	OS	1.971	0.909-4.160	0.0843
	Messager M^33^	56	192	OS	2.47	1.21-5.01	0.012
				DFS	2.85	1.54-5.26	0.001
	Noble F^26^	138	132.36	OS	1.002	1.000-1.005	0.056
				DFS	1.000	0.997-1.003	0.841
	Tianxing G^56^	129	-	OS	1.396	0.843-2.311	0.194
	Urabe M^59^	87	con	OS	1.01	0.86-1.19	0.90
				DFS	0.97	0.84-1.13	0.73
	Wang Y^60^	215	130.8	OS	1.256	0.905-1.742	^b^0.173
	Yuan D^23^	327	150	OS	1.284	0.897-1.838	^b^0.172
			300	OS	1.398	0.872-2.241	^b^0.164
			150	DFS	1.338	0.979-1.829	^b^0.068
			300	DFS	1.352	0.887-2.062	^b^0.161
	Zhang JW^27^	611	124.4	CSS	1.00	1.00-1.00	-
	Zhang L^21^	355	171	OS	1.124	0.789-1.062	0.517
	Zhou WJ^35^	309	96.960	OS	1.188	0.795-1.775	0.402
LMR	Cong X^19^	129	3.25	OS	0.820	0.576-1.167	0.271
	Urabe M^59^	87	con	OS	0.98	0.91-1.06	0.64
				DFS	0.98	0.92-1.06	0.68
	Zhang JW^27^	611	0.223	CSS	2.68	0.85-8.43	0.092
	Zhou WJ^35^	309	0.201	OS	1.604	1.071-2.402	0.022
SII	Cong X^19^	129	451	OS	1.040	0.668-1.618	0.863
	Jomrich G^40^	320	644	OS	-	-	<0.001
				DFS	-	-	<0.001
PLT	Bando H^36^	14	150	OS	-	-	0.76
	Chau I^38^	248	median	OS	0.955	0.839-1.086	^b^0.482
	Jagadesham VP^34^	105	275	MS	-	-	^b^0.425
	Noble F^26^	138	226	OS	1.000	0.997-1.003	0.837
				DFS	1.000	0.997-1.002	0.761
	Yuan D^23^	327	-	OS	1.045	0.835-1.308	^b^0.701
				DFS	1.033	0.846-1.260	^b^0.752
NE	Fuchs CS^37^	-	-	OS	1.52	1.17-1.99	<0.0001
	Noble F^26^	138	4	OS	-	-	0.811
				DFS	1.096	0.972-1.237	0.136
	Yuan D^23^	327	-	OS	1.110	0.901-1.368	^b^0.328
				DFS	1.184	0.985-1.424	^b^0.073
LY	Fuchs CS^37^	-	-	OS	^c^1.31	1.05-1.63	0.0015
	Noble F^26^	138	1.7	OS	0.885	0.687-1.139	0.342
				DFS	1.036	0.845-1.271	0.731
	Yuan D^23^	327	-	OS	0.838	0.648-1.083	^b^0.177
				DFS	0.810	0.650-1.011	^b^0.062
WBC	Chau I^38^	248	-	OS	-	-	0.06
	Noble F^26^	138	-	OS	1.074	0.982-1.175	0.118
				DFS	1.063	0.968-1.167	0.200
	Yuan D^23^	327	-	OS	0.977	0.764-1.246	^b^0.850
				DFS	1.027	0.829-1.272	^b^0.807
HGB	Bando H^36^	14	100 g/l	OS	-	-	^b^0.127
	Chau I^38^	248	110 g/l	OS	-	-	0.011
	Han WX^48^	101	120 g/l	OS	1.000	0.527-1.899	1.000
	Jomrich G^43^	314	-	OS	0.98	0.90-1.06	0.591
				DFS	0.99	0.92-1.07	0.775
	Larsen AC^47^	170	-	OS	-	-	^b^-
	Tianxing G^56^	129	-	OS	-	-	^b^0.095
	Zhang L^21^	355	120 g/l	OS	0.943	0.671-1.318	0.730
	Zhu Z^41^	239	130 g/l	OS	0.689	0.501-0.946	0.021
MCV	Jomrich G^43^	314	-	OS	1.05	1.03-1.08	<0.001
				DFS	1.05	1.03-1.08	<0.001
MCH	Jomrich G^43^	314	-	OS	1.14	1.07-1.22	<0.001
				DFS	1.12	1.05-1.20	<0.001
MCHC	Jomrich G^43^	314	-	OS	1.17	1.07-1.28	0.001
				DFS	1.17	1.07-1.27	<0.001
RDW	Jomrich G^43^	314	-	OS	0.98	0.93-1.04	0.538
				DFS	0.99	0.94-1.05	0.794
HCT	Cao HL^45^	156	-	OS	^c^5.353	3.419-8.380	<0.001

EJC: esophagogastric junction cancer; NLR: neutrophil-lymphocyte ratio; PLR: platelet-lymphocyte ratio; LMR: lymphocyte-monocyte ratio; SII: systemic immune-inflammation score; PLT: platelet; NE: neutrophil count; LY: lymphocyte count; WBC: white blood cell; HGB: hemoglobin; MCV: mean corpuscular volume; MCH: mean corpuscular hemoglobin; MCHC: mean corpuscular hemoglobin concentration; RDW: red blood cell distribution width; HCT: hematocrit; OS: overall survival; DFS: disease-free survival; CSS: cancer-specific survival; TTP: time to progression; MS: median survival; con: continuous variable ^a^ statistical significance in univariate analysis; ^b^ no statistical significance in univariate analysis; ^c^ the HR of low level; ^d^ not included in the multivariate analysis Note: the units for PLT, NE, LY and WBC are 10^9^/l; the unit for RBC is 10^12^/l.

**Table 2:** Blood-based biochemical and coagulation parameters in EJC prognosis

Variables	Authors	Number of EJC patients	Cutoff Values	Survival Types	Hazard Ratio	95% Confidence Interval	*P-*Value
ALB	Bando H^36^	14	35 g/l	OS	-	-	<0.001
	Cao HL^45^	156	35 g/l	OS	^c^1.907	1.058-3.438	0.032
	Chau I^38^	248	median	OS	0.686	0.597-0.790	^a^<0.0001
	Custodio A^20^	155	LLN	-	-	-	-
	Fuchs CS^37^	-	-	OS	^c^1.33	1.07-1.65	0.0006
	Han WX^48^	101	40 g/l	OS	0.945	0.469-1.903	0.874
	Jomrich G^46^	155	35 g/l	OS	0.52	0.33-0.82	0.005
				DFS	0.51	0.33-0.80	0.004
	Larsen AC^47^	170	-	OS	-	-	^b^-
	Noble F^26^	138	35 g/l	OS	-	-	0.137
				DFS	0.957	0.919-0.997	0.034
	Tianxing G^56^	-	42 g/l	OS	-	-	^b^0.725
	Zhang L^21^	355	40 g/l	OS	-	-	0.061
	Zhu Z^41^	239	40 g/l	OS	-	-	^b^0.946
Pre-ALB	Han WX^48^	101	200 g/l	OS	0.494	0.271-0.901	0.021
	Zhang L^21^	355	180 g/l	OS	0.428	0.310-0.592	0.000
BIL	Custodio A^20^	155	ULN	-	-	-	-
ALP	Chau I^38^	248	100 U/l	OS	1.412	1.136-1.755	<0.0001
	Custodio A^20^	155	ULN	-	-	-	-
	Fuchs CS^37^	-		OS	1.28	1.03-1.60	0.0030
LDH	Bando H^36^	14	ULN	OS	-	-	^a^<0.001
	Custodio A^20^	155	ULN	-	-	-	-
	Fuchs CS^37^	-		OS	1.31	1.05-1.63	0.0019
	Larsen AC^47^	170	-	OS	3.03	1.54-5.94	0.001
AST	Fuchs CS^37^	-	-	OS	1.37	1.06-1.76	0.0014
Na	Chau I^38^	248	median	OS	0.721	0.621-0.837	^a^<0.0001
	Fuchs CS^37^	-	-	OS	^c^2.04	1.54-2.71	<0.0001
Ca	Chau I^38^	248	median	OS	1.005	0.856-1.178	^b^0.956
GPS	Cui Y^51^	332	1	OS	2.32	1.69-3.20	<0.001
				DFS	2.36	1.73-3.22	<0.001
			2	OS	5.08	3.01-8.57	<0.001
				DFS	3.01	1.71-5.29	<0.001
	Kudou K^49^	59	1	OS	3.758	1.556-8.234	^a^0.0047
	Jagadesham VP^34^	105	1	MS	1.58	0.62-4.06	0.337
mGPS	Jomrich G^46^	155	1+2	OS	1.72	1.10-2.67	0.017
			1+2	DFS	1.65	1.08-2.50	0.0195
	Park JH^52^	163	1/2	OS	1.24	1.03-1.49	0.021
	Urabe M^59^	87	1	OS	1.08	0.64-1.70	^b^0.093
			2	OS	2.11	1.08-3.69	^b^0.093
			1	DFS	1.07	0.65-1.66	0.081
			2	DFS	0.49	0.25-0.89	0.081
CRP/ALB	Kudou K^49^	59	0.1	OS	2.378	1.025-5.249	0.0439
Fib	Cong X^19^	129	3.09 g/l	OS	2.598	1.851-3.645	^a^<0.001
	Jagadesham VP^34^	105	4.9 μmol/l	MS	-	-	^d^0.005
	Tianxing G^56^	129	3.09 g/l	OS	1.083	0.696-1.684	0.724

EJC: esophagogastric junction cancer; ALB: albumin; Pre-ALB: pre-albumin; BIL: bilirubin; ALP: alkaline phosphatase; LDH: lactate dehydrogenase; AST: aspartate aminotransferase; Na: sodium; Ca: calcium; GPS: Glasgow prognostic score; mGPS: modified GPS; Fib: fibrinogen; LLN: lower limit of normal; ULN: upper limit of normal; OS: overall survival; DFS: disease-free survival; MS: median survival ^a^ statistical significance in univariate analysis; ^b^ no statistical significance in univariate analysis; ^c^ the HR of low level; ^d^ not included in the multivariate analysis

**Table 3:** Combination of hematologic, biochemical and coagulate parameters in EJC prognosis

Variables	Authors	Number of EJC Patients	Cutoff Values	Survival Types	Hazard Ratio	95% Confidence Interval	*P-*Value
CONUT score	Kudou K^49^	59	3	OS	4.749	2.146-10.09	^a^0.0003
F-NLR	Cong X^19^	129	1	OS	1.921	1.124-3.283	0.017
			2	OS	2.764	1.559-4.900	0.001
	Tianxing G^56^	129	-	OS	1.730	1.173-2.551	0.006
PNI	Han WX^48^	101	51	OS	0.751	0.372-1.518	0.426
	Noble F^26^	138	47.50	OS	-	-	0.323
				DFS	0.979	0.950-1.009	0.165
	Urabe M^59^	112	con	OS	0.62	0.47-0.82	<0.001
				DFS	0.60	0.46-0.78	<0.001
	Zhang L^21^	355	51.3	OS	1.192	0.828-1.715	0.345
AGR/PNI	Wang Y^60^	215	1/2	OS	0.613	0.226-0.923	<0.001

EJC: esophagogastric junction cancer; CONUT score: controlling nutritional status score; F-NLR: combination of fibrinogen concentration and neutrophil-lymphocyte ratio; PNI: prognostic nutritional index; AGR: albumin-to-globulin ratio; OS: overall survival; DFS: disease-free survival; con: continuous variable ^a^ statistical significance in univariate analysis

## Abbreviations

EJC: esophagogastric junction cancer
EJA: esophagogastric junction adenocarcinoma
BE: Barrett's esophagus
NLR: neutrophil-lymphocyte ratio
PLR: platelet-lymphocyte ratio
LMR: lymphocyte-monocyte ratio
SII: systemic immune-inflammation score
PLT: platelet
NE: neutrophil count
LY: lymphocyte count
WBC: white blood cell
HGB: hemoglobin
MCV: mean corpuscular volume
MCH: mean corpuscular hemoglobin
MCHC: mean corpuscular hemoglobin concentration
RDW: red blood cell distribution width
HCT: hematocrit
OS: overall survival
DFS: disease-free survival
CSS: cancer-specific survival
TTP: time to progression
MS: median survival
ALB: albumin
Pre-ALB: pre-albumin
BIL: bilirubin
ALP: alkaline phosphatase
LDH: lactate dehydrogenase
AST: aspartate aminotransferase
Na: sodium
Ca: calcium
GPS: Glasgow prognostic score
mGPS: modified GPS
Fib: fibrinogen
LLN: lower limit of normal
ULN: upper limit of normal
CONUT score: controlling nutritional status score
F-NLR: combination of fibrinogen concentration and neutrophil-lymphocyte ratio
PNI: prognostic nutritional index
AGR: albumin-to-globulin ratio
EB virus: Epstein-Barr virus
